# Heart Rate Variability, Neuromuscular and Perceptual Recovery Following Resistance Training

**DOI:** 10.3390/sports7100225

**Published:** 2019-10-18

**Authors:** Andrew A. Flatt, Liam Globensky, Evan Bass, Brooke L. Sapp, Bryan L. Riemann

**Affiliations:** Biodynamics and Human Performance Center, Department of Health Sciences and Kinesiology, 11935 Abercorn St. Savannah, Georgia Southern University, Savannah, GA 31419, USA; lg06442@georgiasouthern.edu (L.G.); eb07865@georgiasouthern.edu (E.B.); bs12999@georgiasouthern.edu (B.L.S.); briemann@georgiasouthern.edu (B.L.R.)

**Keywords:** autonomic, parasympathetic, cardiovascular, performance, monitoring

## Abstract

We quantified associations between changes in heart rate variability (HRV), neuromuscular and perceptual recovery following intense resistance training (RT). Adult males (*n* = 10) with >1 year RT experience performed six sets to failure with 90% of 10 repetition maximum in the squat, bench press, and pull-down. Changes (∆) from pre- to immediately (IP), 24 and 48 h post-RT were calculated for neuromuscular performance markers (counter-movement jump peak power and mean concentric bench press and squat velocity with load corresponding to 1.0 m∙s^−1^) and perceived recovery and soreness scales. Post-waking natural logarithm of the root-mean square of successive differences (LnRMSSD) in supine and standing positions were recorded pre-RT (5 day baseline), IP and two mornings post-RT. All parameters worsened at IP (*p* < 0.05). LnRMSSD measures were not different from baseline by 24 h. Neuromuscular markers were not different from pre-RT by 48 h. Perceptual measures remained suppressed at 48 h. No significant associations among ∆ variables were observed (*p* = 0.052–0.978). These data show varying timeframes of recovery for HRV, neuromuscular and perceptual markers at the group and individual level. Thus, post-RT recovery testing should be specific and the status of one metric should not be used to infer that of another.

## 1. Introduction

An intense bout of resistance training (RT) alters neuromuscular, cardiovascular, metabolic, endocrine, and immune function to meet physiological demands imposed on the body [1]. Individual responses to RT are impacted by training status and training content variables such as volume, intensity, proximity to muscular failure and repetition velocity [1,2,3]. As the time-course of recovery following RT varies among trainees [4], individualized program-modification based on daily recovery status may be superior to standardized RT prescription [5]. Thus, further investigation into practical RT recovery metrics is required to guide monitoring protocols.

Specific RT performance (one or 10 repetition maximum, RM) takes 24–96 h to return to pre-training values [4,6]. Since RM tests can be time-consuming and potentially delay recovery, non-fatiguing correlates of specific RT performance are appealing alternatives to coaches. Promising non-fatiguing markers of recovery following RT include neuromuscular performance tests such as submaximal barbell velocity [7] and countermovement jump (CMJ) characteristics [8], as well as subjective indicators like perceived recovery and soreness scales [9]. Submaximal barbell velocity can be obtained from specific RT exercises (e.g., back squat, bench press) with decrements in mean concentric velocity at a fixed load relative to baseline reflecting impaired neuromuscular recovery [2]. Decrements in CMJ characteristics are also observed post-RT and have been associated with daily changes in RT performance capacity [8]. Finally, the perceived recovery scale (PRS) has been shown to be sensitive to muscle damaging exercise and associates with creatine kinase concentrations and anaerobic performance indices [9,10].

Altered autonomic nervous system function is an established characteristic of inadequate recovery from training [11]. Vagal markers of heart rate variability (HRV) reflect cardiac-parasympathetic modulation and are suppressed for 12–48 h post-RT [2,6,12,13]. HRV is attractive to coaches because it is a non-invasive physiological marker that can be obtained in only a few minutes with inexpensive mobile applications [14]. HRV-guided RT may reduce the risk of overuse injuries [15] while producing comparable RT adaptations to standardized training in a shorter time-period (5 vs. 7 weeks) [16]. Though HRV and neuromuscular responses follow a similar time course for recovery from a single bout of RT at the group level, correlation analyses have not been performed [6,13]. Therefore, whether RT-induced changes in HRV associate with conventional RT recovery metrics at the individual level remains unclear. In addition, post-RT assessment of HRV has been measured in supine and standing positions [17]. Supine measures reflect passive resting conditions while standing measures represent ANS responsiveness to the mild challenge of orthostasis. Optimal positioning for reflecting recovery status from RT also remains to be established. Therefore, the aims of this study were to (1) quantify associations between changes in HRV, perceptual and neuromuscular performance metrics up to 48 h post-RT and (2) to determine which HRV measurement position (e.g., supine or standing) provides stronger associations with recovery markers.

## 2. Materials and Methods

### 2.1. Participants 

Ten males with >1 year of RT experience participated in this study (age = 24.4 ± 4.5 years; height = 180.7 ± 6.7 cm; weight = 94.8 ± 21.4 kg). The sample consisted of local military and university club rugby players who were free from cardiovascular, metabolic, and orthopedic disorders. All subjects provided written informed consent to participate in the study after learning the benefits and risks of the investigation. Ethical approval for this study was granted by the institutional review board (Protocol H18392).

### 2.2. Procedures

#### 2.2.1. Experimental Design

This was an observational study where HRV, neuromuscular and perceptual recovery markers were obtained pre-, immediately post- (IP), 24, and 48 h post-RT. Associations between changes in HRV and changes in neuromuscular and perceptual markers were quantified. Laboratory visit 1 involved HRV and performance testing familiarization and 10 RM testing. Laboratory visit 2 occurred 5 days after visit 1 so that subjects could obtain a 5-day HRV baseline. The second laboratory visit involved pre-RT perceptual ratings (recovery and soreness scales), neuromuscular performance assessment (CMJ characteristics and submaximal barbell velocities) followed by the interventional RT protocol. Outcome variables were assessed again 10-min post-RT. Laboratory visits 3 and 4 occurred 24 and 48 h post-RT for follow-up assessment of recovery markers. Except for IP, HRV measures were performed post-waking. All laboratory visits occurred between 9 and 11 a.m. 

#### 2.2.2. Heart Rate Variability

On the first laboratory visit, subjects were provided with a Bluetooth heart rate strap (H10, Polar Electro, Kempele, Finland) and were familiarized with performing supine and standing HRV recordings via a cost-free mobile application (Elite HRV Version 4.2 Ashville, NC, USA) [18]. The application settings were adjusted so that HRV recordings automatically included a 1-min R-R interval recording preceded by a 1-min stabilization period [19,20]. Subjects were instructed to remain quiet, still, and breathe naturally during recordings. Over the subsequent 5 days, HRV was recorded after waking and averaged to establish baseline. HRV was recorded again IP, and post-waking 1 and 2 days following the interventional RT protocol for comparison to baseline. Subjects were asked to maintain their daily routines throughout the baseline period and to abstain from RT within 72 h of the RT protocol. R-R interval files were exported via email to the researchers for R-R filtering using Kubios software (Version 3.0.2, Kuopio, Finland) [21]. The natural logarithm of the vagal-related root-mean square of successive R-R interval differences (LnRMSSD) was recorded as the HRV index for analysis in accordance with recent recommendations [22]. Ultra-short (i.e., 60-s) LnRMSSD measures have been shown to be no different from criterion 5-min measures, require only a brief stabilization period, and are sensitive to training-induced changes [19,20].

#### 2.2.3. Ten Repetition Maximum 

Ten RM for the squat, bench press, and latissimus dorsi pull-down were assessed on the first laboratory visit following HRV and performance testing familiarization. The warm-up consisted of 10 repetitions with 50% and 70% of their estimated 10 RM, followed by a 10 RM attempt. Load was adjusted by 2.5–5 kg until a 10 RM was obtained. A 3-min rest was provided between attempts. Strict technique was enforced during all repetitions. During the squat, a neutral or slightly hyperextended lumbar spinal position was maintained while the hips, knees, and ankles flexed to a minimum depth where the femur was parallel with the floor. For bench press, the barbell touched the chest without bouncing during the eccentric phase and elbows were fully extended during the concentric phase. The posterior head, upper-back, and hips remained in contact with the bench while feet remained in contact with the floor for the duration of a set. Pull-downs required that the bar touched the clavicle during the concentric phase and that elbows were fully extended during the eccentric phase with minimal movement of the trunk. 

#### 2.2.4. Neuromuscular Performance

Subjects were familiarized with the neuromuscular performance testing protocol at laboratory visit 1, following HRV familiarization. Performance values were subsequently recorded immediately pre-, IP, 24, and 48 h post-RT protocol. A standardized warm-up involving 5 min (100 W at 80–90 r∙m^−1^) on a cycle ergometer (928 E, Monark, Vansbro, Sweden), followed by dynamic stretches for the upper and lower limbs was performed before testing. CMJ analysis consisted of three warm-up jumps performed at 50%, 70%, and 90% of maximum effort, followed by three maximal jumps with 60 s rest between attempts. To perform the CMJ, subjects were instructed to fix their hands on the hips, flex the hips and knees to a self-selected depth and jump as high as possible. Ground reaction forces (GRF) during the CMJ were collected using a force plate (OR6, AMTI, Watertown, MA, USA) sampling at 1000 Hz using The Motion Monitor acquisition software (Version 9, IST, Chicago, IL, USA). Data reduction procedures were conducted using Matlab (Mathworks, Natick, MA, USA). First, GRF were filtered using a zero-phase lag Butterworth filter (50-Hz cutoff). Vertical velocity of the subject’s total body center of mass was calculated by subtracting body weight from the vertical GRF-time curve, dividing by body mass, and integrating with respect to time using the trapezoidal rule [23]. Vertical mechanical power was calculated as the product of vertical GRF and vertical velocity. The peak power (PP) attained during the countermovement phase of the jump was used for analysis due to its sensitivity to RT-induced changes [8] and acceptable reliability (CV = 2.7%) [24].

Average concentric barbell velocity for the back squat and bench press were collected after CMJ assessment with a linear position transducer (Tendo™ Weightlifting Analyzer, Tendo Sports, Trencin, Slovakia) [25] in a power rack (ProMaxima, Houston, TX, USA). Subjects were instructed to pause for 1 s when the femur was parallel with the floor during the squat and when the barbell touched the chest during the bench press. The concentric phase was performed as fast as possible. Progressive attempts were performed with 90-s inter-set rest until the maximal loads corresponding to 1.0 m·s^−1^ (V1.0) were obtained for each movement (squat first followed by bench press) [2]. When V1.0 was obtained with a given load, a second attempt was provided with an additional 2.27 kg. If V1.0 was not attained on this attempt, the original V1.0 load was recorded for analysis. Maximal velocities were again obtained with the same V1.0 loads at IP, 24, and 48 h post-RT for comparison to pre-RT values. V1.0 barbell measures are sensitive to training-induced changes and have shown acceptable absolute reliability (CV = 4.1%) [26].

#### 2.2.5. Perceptual Markers

Perceived recovery (PRS) and soreness scales (PSS) [10] were obtained pre-, IP, 24, and 48 h post-RT using standardized 0–10 scales. For PRS, 0 = “poorly recovered” and 10 = “very well recovered” [10]. For PSS, 0 = “normal, not sore at all” and 10 = “severely sore” [10].

#### 2.2.6. Resistance Training Protocol

The RT protocol was performed immediately after acquisition of pre-training neuromuscular performance and perceptual measures at laboratory visit 2. The protocol consisted of six sets to momentary muscular failure for the squat, bench press, and latissimus dorsi pulldown with 90% of 10 RM. Subjects were given 90 s rest between sets and 2 min rest between exercises. RT volume load (total repetitions × load in kg, summed for each movement) and exercising heart rate (HRex, mean and peak) were recorded to characterize the external mechanical and internal cardiovascular load, respectively (Table 1). HRex was obtained using the Polar H10 Bluetooth HR monitor and mobile application (Polar Beat Version 2.6.2, Polar Electro, Kempele, Finland).

### 2.3. Statistical Analysis

Data normality was assessed with Shapiro–Wilks tests. Repeated measures analysis of variance was used to compare HRV and CMJ across time. Tukey tests were used for post-hoc analyses. Friedman’s test (non-parametric) was used for the non-normally distributed (*p* < 0.05) V1.0 and perceptual variables. Wilcoxon Signed Rank tests with Bonferroni *p*-value adjustments were used for post-hoc analyses. Cohen’s d effect sizes (ES) for normally distributed variables and adjusted Cohen’s d ES for non-normally distributed variables were used to assess the magnitude of differences among variables [27]. Qualitative thresholds for the ES were <0.20 = trivial; <0.59 = small; <1.19 = moderate; <2.0 = large; ≥2.0 = very large [28]. Changes (∆, post-measure − pre-measure) in outcome variables were quantified for all recovery markers at IP, 24, and 48 h post-RT relative to pre-RT or baseline. Pearson’s correlations were used to quantify relationships between ∆LnRMSSD, ∆perceptual, and ∆neuromuscular performance metrics and total volume load. Qualitative thresholds for correlations were <0.10 = trivial; <0.30 = small; <0.50 = moderate; <0.70 = large; <0.90 = very large; ≥0.90 = nearly perfect [29]. Statistical significance was set at *p* < 0.05. Statistical procedures were performed using JASP (Version 0.10.2, University of Amsterdam, Amsterdam, The Netherlands) and JMP (Version 13, SAS Institute, Cary, NC, USA). 

## 3. Results

### 3.1. Model Effects

**Figure 1:**
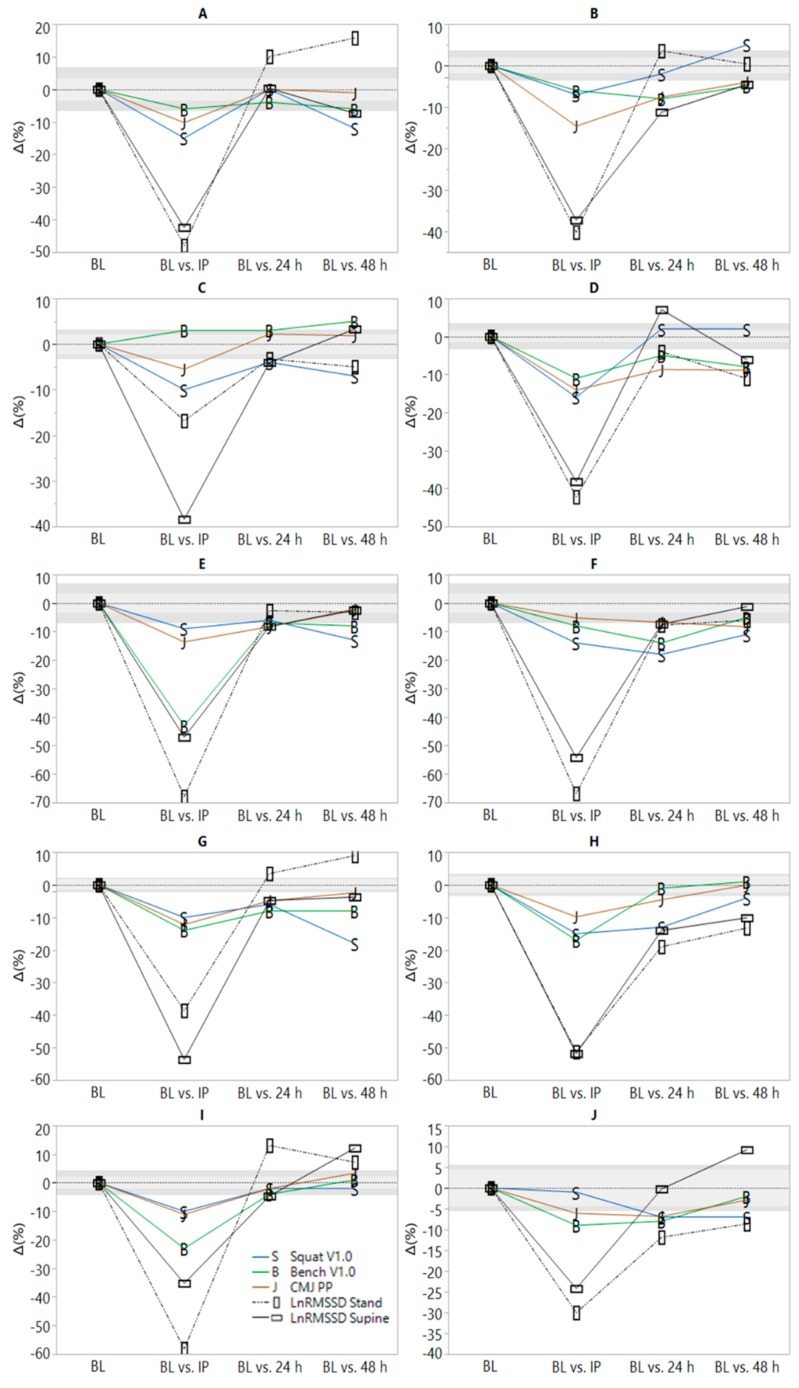
Individual time-course of recovery following resistance training (RT). BL = baseline; IP = 10 min post-RT. Light and dark gray shaded areas represent smallest worthwhile change thresholds (0.5 × intra-individual BL coefficient of variation) [30] for supine and standing natural logarithm of the root mean square of successive differences (LnRMSSD), respectively.

Significant model effects were observed for all parameters (Table 2). All variables worsened at IP relative to pre-RT. LnRMSSD measures were not different from baseline by one morning post-RT. CMJ, bench press, and squat V1.0 were not different from pre-RT by 48 h. PRS and PSS remained suppressed relative to pre-RT at 48 h. Values and ES are displayed in Table 2. Substantial inter-individual variation in recovery responses were observed and can be viewed in Figure 1. 

### 3.2. Associations

No significant associations were observed between variables (*p* values ranged from 0.052–0.978). Correlation coefficients (r) are presented in Table 3. 

## 4. Discussion

We investigated the association between changes in HRV, perceptual and neuromuscular recovery metrics up to 48 h following an intense bout of RT. The main finding was that recovery markers showed varying timeframes of recovery at the group and individual levels. Additionally, ∆LnRMSSD from the standing position provided stronger associations (i.e., greater r values) than supine measures with several neuromuscular and perceptual recovery markers, although relationships were inconsistent across time and were all non-significant (*p* < 0.05).

Previous investigations concerning HRV responses ~24 h post-RT have found no change [2] or significant reductions from baseline [6,12,17]. Chen et al. monitored HRV (seated position), perceived soreness, and 1RM performance immediately pre- and 24, 48, and 72 h post-RT in high-level weightlifters (*n* = 7 males) [6]. High frequency spectral power (HF, a parasympathetic HRV index) was suppressed at 24 h post-RT (*p* < 0.05) but recovered to baseline or above thereafter [6]. Schneider et al. observed HRV responses in athletes to a 6-day RT overload microcycle involving two full-body RT sessions per day (3–4 exercises per session, four sets of six repetitions with 85% of 1RM) [17]. Decrements in supine, but not standing LnRMSSD were observed (ES ~ −0.50) after day 1 and remained suppressed until 48 h post-microcycle [17]. Both supine and standing LnRMSSD were not different (*p* > 0.05) from baseline by 24 h post-RT in the current study. Methodological differences between studies may account for the conflicting LnRMSSD recovery timeframes. For example, Chen et al. imposed a 10-day detraining period prior to the interventional RT protocol [6]. Exposure to intense RT after training cessation of this duration likely resulted in greater cardiac-autonomic disturbance than would be expected with greater training frequency [2]. Supporting this postulation, nocturnal HRV was no different from baseline following an RT protocol (three sets to failure with 8RM load for squat and bench press) in nine resistance-trained men, preceded by only four days of training cessation [2]. Finally, Schneider et al. prescribed both a.m. and p.m. training sessions, providing less recovery time prior to post-waking HRV assessment. 

Few studies have examined associations between ∆HRV, ∆neuromuscular, and ∆perceptual recovery metrics 24–48 h post-RT. Chen at al. observed no significant reductions in 1RM at 24 h post-RT despite decrements (*p* < 0.05) in HF and muscular soreness [6]. HF and 1RM performance peaked simultaneously at 72 h post-RT, leading the authors to conclude that HRV may reflect neuromuscular recovery status. However, correlations between ∆HRV, ∆performance, and ∆subjective markers were not reported [6]. Schneider et al. found significant reductions in maximal upper but not lower-body isometric force and reported weak or inconsistent associations with ∆LnRMSSD following an RT overload microcycle [17]. Gonzalez-Badillo et al. found significant reductions in CMJ height but not barbell squat or bench press V1.0 at 24 h post-RT [2]. No significant associations were found with nocturnal ∆LnRMSSD and standing measures were not assessed [2]. In the current study, supine and standing LnRMSSD responses followed a similar trend at the group level (Table 2), but relative changes at the individual level were inconsistent (Figure 1). Qualitatively large (r ≥ 0.5) but non-significant associations (*p* < 0.05) were observed between ∆LnRMSSD standing, ∆squat V1.0, ∆PRS, and ∆PSS in the current study at 24 h post-RT. Associations between seated upright LnRMSSD and perceptual recovery markers have been previously reported in sprint-swimmers [31]. However, the inconsistency in associations between ∆LnRMSSD and ∆neuromuscular markers at 24 and 48 h post-RT are in agreement with a previous case-comparison showing that soccer players demonstrated impaired post-submaximal exercise LnRMSSD but stable CMJ performance (or vice-versa) throughout a competitive training camp [22].

A novel feature of the current investigation pertains to the graphical display of individual timeframes of post-RT recovery. Figure 1 shows considerable inter-individual heterogeneity in recovery responses both within and between markers representative of different (psycho) physiological systems (i.e., cardiac-autonomic, neuromuscular and perceived psychological). This suggests that not only should practitioners avoid using recovery status of one system (i.e., cardiac-autonomic) to infer that of another (i.e., neuromuscular or perceived psychological), but that specificity of testing must also be considered for parameter-selection within a given system. This is because varying timeframes of recovery within and between subjects can be observed for the two HRV measurement positions, the three neuromuscular performance tests, and the two perceptual indices. In other words, cardiac-autonomic recovery is position-dependent and neuromuscular recovery is movement (e.g., CMJ vs. squat) and body-segment (e.g., upper- vs. lower-body) dependent. Thus, the complex nature of recovery likely requires that multiple and specific recovery metrics are needed to ascertain the status of a given system due to differences in their physiological responses to RT. For example, post-exercise LnRMSSD responses are influenced by lactate and metabolite accumulation (i.e., metaboreflex stimulation), elevated body temperature, circulating catecholamines, and disturbed fluid balance [32]. Restoration of LnRMSSD to baseline following a single bout of intense exercise generally requires ≤48 h [32]. Neuromuscular responses are influenced by central (e.g., inhibited central motor drive due to metabolite activation of group III and IV afferent fibers) and peripheral factors (e.g., muscle damage and inflammation) [33]. Delayed muscle soreness [34] and neuromuscular performance [35] often require ≥48 h to return to baseline at the individual level and may explain why neuromuscular and perceptual recovery timeframes may exceed that of cardiac-autonomic recovery following intense RT.

This study was limited by sample size, RT history of the subjects, utilization of smartphone-derived HRV measures, lack of biochemical analyses, and inclusion of only submaximal performance markers. Future research should investigate whether supine or standing HRV responses to chronic RT can be useful for reflecting adaptations, aid in avoidance of overreaching, and minimizing musculoskeletal overuse injuries.

## 5. Conclusions

HRV, neuromuscular and perceptual markers demonstrated varying timeframes of recovery at the group and individual levels following intense RT. Changes in standing LnRMSSD demonstrated stronger associations (i.e., higher r values) than supine measures with changes in other recovery metrics, although relationships were inconsistent across time and were all non-significant. Practitioners are therefore encouraged to be specific with their post-RT recovery testing and not use the status of a given physiological system (e.g., cardiac-autonomic) to infer the status of another (e.g., neuromuscular or perceived psychological). Each of these appear to be independent recovery markers that may offer unique strengths in informing on differing aspects of post-RT recovery and adaptation. 

## Figures and Tables

**Table 1:** Internal and external resistance training load parameters reported as mean ± standard deviation.

External Load	Load (kg)	Repetitions	Volume Load (kg)
Squat	94.7 ± 12.7	62.7 ± 10.3	5952 ± 1310
Bench Press	81.1 ± 11.6	38.8 ± 4.5	3122 ± 480
Pull-Down	50.5 ± 6.6	42.2 ± 7.6	2098 ± 282
Total Volume	-	-	11,173 ± 1441
Internal Load	Peak (b∙min^−1^)	Mean (b∙min^−1^)	-
HRex	177.5 ± 14.7	136.4 ± 19.2	-

HRex = exercising heart rate. Total Volume = total repetitions × load in kg and summed for each movement.

**Table 2:** Pre- and post-training mean ± standard deviation or median (inter-quartile range) for recovery metrics.

Recovery Metric	Pre/BL	IP	24 h P	48 h P	Model Effect (*p*)
Supine LnRMSSD	4.38 ± 0.74	2.32 ± 0.48 *^V^	4.18 ± 0.81 ^S^	4.31 ± 0.59	<0.0001
Standing LnRMSSD	3.45 ± 0.32	1.83 ± 0.56 *^V^	3.38 ± 0.47	3.40 ± 0.48	<0.0001
CMJ Peak Power (W)	4877 ± 432	4375 ± 404 *^M^	4636 ± 321 *^M^	4754 ± 427 ^S^	<0.001
Squat V1.0 (m·s^−^^1^)	1.00 (0.00)	0.90 (0.07) *^V^	0.95 (0.07) *^V^	0.93 (0.11) ^V^	0.002
Bench Press V1.0 (m·s^−^^1^)	1.00 (0.00)	0.90 (0.13) *^V^	0.94 (0.05) *^V^	0.95 (0.09) ^L^	0.002
Perceived Soreness (au)	1.00 (1.25)	5.50 (4.00) *^V^	5.50 (3.25) *^V^	6.50 (3.00) *^V^	<0.001
Perceived Recovery (au)	8.50 (2.00)	4.00 (3.00) *^V^	5.00 (1.50) *^V^	6.50 (3.25) *^V^	0.001

BL = baseline; LnRMSSD = natural logarithm of the root-mean square of successive R-R interval differences; CMJ = countermovement jump; IP = immediately post-training; 24 h P = 24 h post-training; 48 h P = 48 h post-training; V1.0 = mean concentric velocity at 1.0 meter per second. * = different from Pre/BL (*p* < 0.05). ^S^ = small effect size ^M^ = Moderate effect size ^L^ = large effect size ^V^ = Very large effect size. Note that effect sizes represent the magnitude of change in a variable relative to baseline or pre-RT and are independent of statistical significance.

**Table 3:** Correlation coefficients for changes (∆) in the natural logarithm of the root-mean square of successive R-R interval differences (LnRMSSD), ∆neuromuscular and ∆perceptual recovery metrics relative to pre-training or baseline values and total volume load.

Recovery Metric	∆LnRMSSD Supine (%)		∆LnRMSSD Standing (%)	
	24 h P	48 h P	24 h P	48 h P
∆Squat V1.0 (%)	r = 0.58	r = 0.04	r = 0.63	r = −0.38
∆Bench Press V1.0 (%)	r = −0.06	r = 0.45	r = −0.08	r = −0.23
∆CMJ Peak Power (%)	r = 0.04	r = 0.39	r = 0.35	r = 0.36
∆Perceived Recovery (au)	r = −0.01	r = −0.36	r = 0.50	r = 0.06
∆Perceived Soreness (au)	r = −0.01	r = −0.37	r = −0.58	r = −0.47
Total Volume Load (kg)	r = 0.16	r = −0.10	r = −0.41	r = −0.47

LnRMSSD = natural logarithm of the root-mean square of successive R-R interval differences; CMJ = countermovement jump.
